# A computational method for the coupled solution of reaction–diffusion equations on evolving domains and manifolds: Application to a model of cell migration and chemotaxis

**DOI:** 10.1016/j.jcp.2015.12.038

**Published:** 2016-03-15

**Authors:** G. MacDonald, J.A. Mackenzie, M. Nolan, R.H. Insall

**Affiliations:** a Department of Mathematics and Statistics, University of Strathclyde, Glasgow, G1 1XH, United Kingdom; b The Beatson Institute for Cancer Research, Garscube Estate, Switchback Road, Glasgow, G61 1BD, United Kingdom

**Keywords:** Reaction–diffusion, Bulk–surface equations, Cell migration, Chemotaxis, Evolving finite elements, ALE methods, Moving mesh methods

## Abstract

In this paper, we devise a moving mesh finite element method for the approximate solution of coupled bulk–surface reaction–diffusion equations on an evolving two dimensional domain. Fundamental to the success of the method is the robust generation of bulk and surface meshes. For this purpose, we use a novel moving mesh partial differential equation (MMPDE) approach. The developed method is applied to model problems with known analytical solutions; these experiments indicate second-order spatial and temporal accuracy. Coupled bulk–surface problems occur frequently in many areas; in particular, in the modelling of eukaryotic cell migration and chemotaxis. We apply the method to a model of the two-way interaction of a migrating cell in a chemotactic field, where the bulk region corresponds to the extracellular region and the surface to the cell membrane.

## 1 Introduction

Coupled bulk–surface problems arise in many areas of engineering and the applied and natural sciences. Examples include crystal growth [29], interfacial fluid flows using soluble and insoluble surfactants [7], [27], proton diffusion along biological membranes [34] and cell signalling [47].

Our motivation for the development of a method for coupled problems on evolving domains comes from the study of eukaryotic cell migration and chemotaxis. Chemotaxis, in particular, is essential during embryonic development, immune cell function, and cancer metastasis. Eukaryotic cells typically crawl by protruding pseudopods, which are dynamic structures based on actin fibres, at the front of the cell [26]. Actin is a globular protein which spontaneously polymerises into linear filaments that make up a large fraction of the cell's cytoskeleton. The key step limiting actin polymerisation is the slow initiation of new filaments. Actin assembly can therefore be stimulated by “nucleating factors” which generate new actin filaments. In most cells, actin filaments are concentrated just beneath the cellular membrane and crosslinked into a relatively rigid cortex. Motor proteins such as myosin II bind to actin filaments in the cortex, crosslinking and contracting them, causing cortical tension and mechanical resistance, which together are key determinants of cells' overall behaviour. New actin polymerisation occurs between the cortex and the membrane, giving rise to a pressure pushing the cell membrane outward at the pseudopod; in other regions, cortical tension pulls along the remainder of the cell body. Together these processes lead to cell movement.

In [40] we developed a preliminary model of cell migration and chemotaxis where we only considered processes taking place on or close to the cell membrane. The model was based on a system of three reaction–diffusion equations posed on an evolving curve in two dimensions. An evolving surface finite element method was used to approximate the solution of the PDEs and a level set method was used to move the cell boundary. The method was later made more robust and more efficient by replacing the level set method by a parameterised finite element approach [39]. The model was shown to predict many aspects of real cell behaviour such as cell polarisation, persistent random walk migration in the absence of external signals and directed migration in the presence of a gradient in an external chemotactic field [41].

The modelling of a number of important biological processes however also requires the ability to solve model equations in the extracellular and intracellular domains which are then coupled to equations posed on the cell membrane. It is also well appreciated that migrating cells have the ability to shape external chemotactic fields through the use of membrane-bound enzymes that degrade the ligand field and by the self-secretion of chemoattractants that allow signal relay to neighbouring cells [50]. The modelling of these important effects require the computational ability of solve partial differential equations on evolving bulk–surface domains.

Some previous computational studies looking at biological signalling have been carried out assuming fixed cell boundaries [36], [30], [42]. Methods have also been proposed to treat the deforming cell shape; for a recent review see [18]. For example, a phase-field approach has been used to model the combined effect of intracellular actin-flow, cell adhesions and morphology on cell motility [48]. Normally phase-field methods use isotropic stationary meshes which makes their implementation relatively straightforward. However, as the phase-field is smoothed over a small width of size *ε*, the computational mesh needs to be fine enough to resolve this spatial scale which can make the method computationally expensive unless some form of local mesh adaptation is used. Another well known embedding method is the level set method (LSM) [44]. This approach uses an evolution equation to move a signed distance function; the zero level set of this function is then identified as the moving cell boundary. LSMs have been used successfully in cell motility models [49], [54]. The disadvantage of LSMs is the requirement to maintain a high quality signed distance function which can become difficult if the evolving cell morphology becomes complicated. Bulk–surface reaction–diffusion systems on stationary domains have also been addressed using a simple point cloud method in [31]. An analysis of a finite element method for a steady state bulk–surface problem is presented in [13]. Analysis and computations of the diffusion-driven instability properties of coupled bulk–surface reaction–diffusion systems on stationary domains is presented in [32], [33].

To approximate the solution of bulk–surface systems of equations we will use an Arbitrary Lagrangian–Eulerian (ALE) approach. Traditionally, ALE methods have been used to solve problems on moving domains using a reformulation of the original problem with respect to an alternative reference frame rather than the standard fixed Eulerian frame. For fluid dynamics problems one could decide to use a Lagrangian transformation to follow the fluid flow. More generally however, there may be no obvious or preferred reference frame, and if the domain moves in time one may simply be satisfied with a transformation from a fixed stationary domain $\Omega_{c}$ onto the physically evolving domain Ω(t). The ALE formulation was introduced for this purpose and it has been used successfully to tackle a number of physical applications such as fluid–structure interaction systems (see [24], [11]). In this paper we will use a conservative finite element ALE scheme. An advantage of the finite element framework is it allows the natural incorporation of flux boundary conditions linking the solution components in the bulk and surface domains. Another potential advantage of the ALE approach is the ability to accommodate arbitrary mesh movements which are not necessarily Lagrangian. This may help the robustness, accuracy and efficiency of the method with fewer instances of remeshing being required.

Crucial to the success of the front-tracking approach used here is the robust generation of cell body-fitted meshes. For this purpose we use an adaptive moving mesh procedure based on the solution of a moving mesh partial differential equations (MMPDEs) [9], [19], [4], [8], [23]. Although adaptive moving meshes have been used successfully to solve a range of physical problems [5], [51], [6], to our knowledge they have not been applied to the generation of meshes for curves evolving according to a geometric evolution law. An important contribution of this paper therefore is the development of an MMPDE method for the tangential motion of grid nodes along a curve which is moving with a specified normal velocity. The proposed method is motivated by the approach of Pan and Wetton [45], who devised a finite difference scheme for the generation of meshes which equidistribute the arc-length between grid nodes. The new method allows a degree of control over the generation of the surface mesh which is then used as known boundary conditions for the generation of the bulk mesh. The additional control of the boundary mesh also adds considerable robustness to the grid generation algorithm leading to far fewer instances for the need of global remeshing due to poor mesh quality.

The layout of this paper is as follows. In the next section we introduce the necessary notation for the description of coupled bulk–surface conservation laws on evolving domains which are then reformulated using a conservative weak ALE formulation. In section 3 we present a moving finite element discretisation of the coupled equation system. We then describe the generation of computational meshes covering the evolving bulk and surface domains in section 4. The complete algorithm is presented in section 5 and applied in section 6 to the simulation of cell migration in a chemotactic field. Finally, we draw some conclusions and directions for further research in section 7.

## 2 Model system equations

**Fig. 1:**
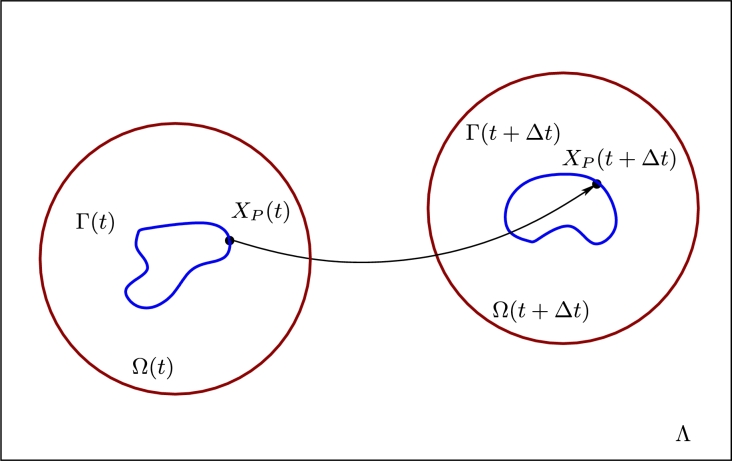
We consider the simulation of a motile cell through a fixed lab frame of reference Λ. The cell membrane is denoted by Γ(*t*) and the extracellular region close to the cell is denoted by Ω(*t*). After a time interval of size Δ*t*, the material point located at ***X***_*p*_(*t*) on the cell membrane Γ(*t*) evolves to the new location ***X***_*p*_(*t* + Δ*t*).

The physical layout for the cell migration simulations considered later is shown in Fig. 1. The cell membrane will be denoted by the evolving curve Γ(t). The cell will be assumed to be moving through fixed lab frame of reference Λ. To improve computational efficiency, the governing equations for the extracellular region will only be computed over the time-dependent two-dimensional domain Ω(t), which is centred on the centroid of the cell. We will assume that a material particle *P* located at $X_{p}(t)$ on Γ(t) has velocity ${\overset{˙}{X}}_{p}(t)$. Therefore, we assume that there exists a velocity field ***u*** so that points on Γ(t) evolve with a velocity field ${\overset{˙}{X}}_{p}(t)=u(X_{p}(t),t)$. Let $n=(n_{1},n_{2})$ denote the unit outward normal to Γ(t) and let N(t) be any open subset of $R^{2}$ containing Γ(t). For any function *ζ* which is differentiable in N(t), we define the tangential gradient on Γ(t) by $\nabla_{\Gamma }\zeta =\nabla \zeta -(\nabla \zeta \cdot n)n$, where ⋅ denotes the usual scalar product and ∇*ζ* denotes the usual gradient on $R^{2}$. For a vector function $\zeta =(\zeta_{1},\zeta_{2})\in R^{2}$, the tangential divergence is defined by

$$\nabla_{\Gamma }\cdot \zeta =\nabla \cdot \zeta -\sum_{i=1}^{2}(\nabla \zeta_{i}\cdot n)n_{i}.$$

The Laplace–Beltrami operator on Γ(t) is defined as the tangential divergence of the tangential gradient $\Delta_{\Gamma }\zeta =\nabla_{\Gamma }\cdot (\nabla_{\Gamma }\zeta )$.

Reaction–diffusion systems of the type studied in pattern formation generally exclude cross-diffusion and are only coupled by the reaction kinetics terms. Therefore, we consider the behaviour of a single chemical species with a straightforward generalisation to a system of interacting chemicals. Let us define

(1) $$Q_{T}=\{(x,t)\in R^{3}:x\in \Omega (t),t\in (0,T]\}.$$

Application of Reynolds transport theorem to the equation for mass conservation for a chemical *C*, which diffuses with constant *D*, undergoing reaction at a rate f(c), gives

(2) $$\frac{\partial c}{\partial t}=D\Delta c+f(c),\;(x,t)\in Q_{T},$$

where c(x,t) is the concentration at position x∈Ω(t) at time *t*.

We also consider the evolution of a chemical species $C_{s}$, that resides on the boundary Γ(t). The bulk species *C* will be coupled to $C_{s}$ through the generally nonlinear flux boundary condition

(3) $$\overset{\text{Diffusive flux}}{\overset{︷}{{-D\frac{\partial c}{\partial n}|}_{\Gamma (t)}}}+\overset{\text{Advective flux}}{\overset{︷}{{\left(u\cdot n)c\right|}_{\Gamma (t)}}}=\overset{\text{Rate of surface reaction}}{\overset{︷}{g{\left(c\right|}_{\Gamma (t)},c_{s}),}}$$

where $c_{s}(x,t)$ denotes the concentration of $C_{s}$ at the point x∈Γ(t) and ***n*** is the outward unit normal to Γ(t). At the front of a motile cell (u⋅n)>0, leading to an advective flux onto Γ(t), and at the back of the cell (u⋅n)<0 leading to a flux off of Γ(t). This so-called windshield effect can potentially have a bearing on the ability of highly motile cells to chemotax efficiently in shallow gradients unless some additional mechanisms are employed by the cell such as surface ligand degradation or receptor internalisation [16].

In an analogous manner to the equation for the bulk species, we will assume that the boundary species evolves such that

(4) $$\frac{\partial c_{s}}{\partial t}+\nabla_{\Gamma }\cdot (uc_{s})=D_{s}\Delta_{\Gamma }c_{s}+g{\left(c\right|}_{\Gamma (t)},c_{s})+h(c_{s}),\;(x,t)\in \Gamma (t)\times (0,T),$$

where $D_{s}$ is the boundary diffusion coefficient, $h(c_{s})$ is a surface reaction term.

### 2.1 ALE reformulation

For the reasons mentioned earlier, when the domain is moving a common frame of reference adopted for computational purposes is the Arbitrary Lagrangian Eulerian (ALE) frame. Let $A_{t}$ be a family of bijective mappings, which at each t∈I=[0,T], map points in a reference or computational configuration $\Omega_{c}$ with coordinates ξ=(ξ,η), to points in the current physical configuration Ω(t) with coordinates x=(x,y), so that

(5) $$A_{t}:\Omega_{c}\subset R^{2}\to \Omega (t)\subset R^{2},\;\;x(\xi ,t)=A_{t}(\xi ).$$

The computational configuration could simply be the initial physical configuration Ω(0). We leave the discussion on how to construct the mapping $A_{t}$ to section 4.

For an arbitrary function $g:Q_{T}\to R$, defined on the fixed Eulerian frame, its temporal derivative in the ALE frame is defined as

(6) $${\frac{\partial g}{\partial t}|}_{\xi }:Q_{T}\to R,\;{\frac{\partial g}{\partial t}|}_{\xi }(x,t)=\frac{\partial \overset{ˆ}{g}}{\partial t}(\xi ,t),\;\xi =A_{t}^{-1}(x),$$

where $\overset{ˆ}{g}:\Omega_{c}\times I\to R$ is the corresponding function in the ALE frame; that is $\overset{ˆ}{g}(\xi ,t)=g(x(\xi ,t),t)=g(A_{t}(\xi ),t)$. Taking the time derivative of the ALE mapping defines the ALE velocity ***w*** as

(7) $$w(x,t)={\frac{\partial x}{\partial t}|}_{\xi }(A_{t}^{-1}(x),t).$$

It is important to note that the ALE velocity ***w*** on Γ(t) will, in general, be different from the material velocity ***u***. When w=u the ALE transformation will be purely Lagrangian in nature. To relate the time derivatives with respect to the ALE transformation to the material derivative, a standard application of the chain rule gives

(8) $${\frac{\partial c}{\partial t}|}_{\xi }={\frac{\partial c}{\partial t}|}_{x}+w\cdot \nabla c.$$

The reformulation of (2) in terms of the ALE reference frame therefore takes the form

(9) $${\frac{\partial c}{\partial t}|}_{\xi }-w\cdot \nabla c=D\Delta c+f(c),\;\;\xi \in \Omega_{c}.$$

Similarly, on the boundary the ALE formulation of (4) takes the form

(10) $${\frac{\partial c_{s}}{\partial t}|}_{\xi }+\nabla_{\Gamma }\cdot (uc_{s})-w\cdot \nabla_{\Gamma }c=D_{s}\Delta_{\Gamma }c+g{\left(c\right|}_{\Gamma (t)},c_{s})+h(c_{s}),\;\;\xi \in \partial \Omega_{c}.$$

The ALE reformulated equations (9) and (10) remain coupled through the flux boundary condition (3).

### 2.2 A conservative weak ALE formulation

To construct a weak formulation of (9), we consider a space of admissible test functions defined on the reference domain made of functions $\overset{ˆ}{v}\in H^{1}(\Omega_{c})$. The ALE mapping then defines a set H(Ω(t)) of test functions on the domain Ω(t), as follows:

$$H(\Omega (t))=\left\{v:\Omega (t)\to R:v=\overset{ˆ}{v}\circ A_{t}^{-1},\;\overset{ˆ}{v}\in H^{1}(\Omega_{c})\right\},\;t\in I.$$

A weak formulation of (9) can be obtained using Reynolds transport formula which states that if ψ(x,t) is a function defined on Ω(t), and $V_{t}\subseteq \Omega (t)$ such that $V_{t}=A_{t}(V_{c})$ with $V_{c}\subseteq \Omega_{c}$, then

(11) $$\frac{d}{dt}\int_{V_{t}}\psi (x,t)\;dx=\int_{V_{t}}\left({\frac{\partial \psi }{\partial t}|}_{\xi }+\psi \nabla \cdot w\right)\;dx=\int_{V_{t}}\left({\frac{\partial \psi }{\partial t}|}_{x}+\nabla \psi \cdot w+\psi \nabla \cdot w\right)\;dx.$$

As functions $\overset{ˆ}{v}\in H^{1}(\Omega_{c})$ do not depend on time, then for any v∈H(Ω(t)) we can establish from (11) that

(12) $$\frac{d}{dt}\int_{\Omega (t)}v\;dx=\int_{\Omega (t)}v\nabla \cdot w\;dx$$

and

(13) $$\frac{d}{dt}\int_{\Omega (t)}v\psi \;dx=\int_{\Omega (t)}v\left({\frac{\partial \psi }{\partial t}|}_{\xi }+\psi \nabla \cdot w\right)\;dx.$$

Multiplying (9) by a test function v∈H(Ω(t)), integrating over Ω(t) and the use of (3), (12) and (13) gives the conservative weak form: find *c* such that

(14) $$\begin{array}{c} \frac{d}{dt}\int_{\Omega (t)}cv\;dx-\int_{\Omega (t)}(\nabla \cdot (wc))\;v\;dx+D\int_{\Omega (t)}\nabla c\cdot \nabla v\;dx+\int_{\Gamma (t)}(g-(u\cdot n)c)v\;ds\\ \;=\int_{\Omega (t)}fv\;dx,\;\forall v\in H(\Omega (t)). \end{array}$$

Similarly, on the boundary we have the conservative weak formulation: find $c_{s}$ such that

(15) $$\begin{array}{c} \frac{d}{dt}\int_{\Gamma (t)}c_{s}v_{s}\;ds+\int_{\Gamma (t)}(\nabla_{\Gamma }\cdot ((u-w)c_{s}))\;v_{s}\;ds+D_{s}\int_{\Gamma (t)}\nabla_{\Gamma }c_{s}\cdot \nabla_{\Gamma }v_{s}\;ds\\ \;=\int_{\Gamma (t)}(g+h)v_{s}\;ds,\;\forall v_{s}\in H_{s}(\Gamma (t)), \end{array}$$

where

$$H_{s}(\Gamma (t))=\left\{v_{s}:\Gamma (t)\to R:v_{s}={\overset{ˆ}{v}}_{s}\circ A_{t}^{-1},\;{\overset{ˆ}{v}}_{s}\in H^{1}(\Gamma_{c})\right\},\;t\in I,$$

is the space of test function on Γ(t).

## 3 Moving finite element discretisation

### 3.1 Spatial semi-discretisation

We will assume that, for each t∈[0,T], the physical and reference domains Ω(t) and $\Omega_{c}$ are approximated by polygonal domains $\Omega_{h}(t)$ and $\Omega_{c,h}$, respectively. We will assume that $\Omega_{c,h}$ is covered by a fixed triangulation $T_{h,c}$ with straight edges, so that $\Omega_{c,h}=\cup_{K\in T_{h,c}}K$. The approximation of the boundary domain $\Gamma_{h}(t)$ is chosen to be simply the boundary of $\Omega_{h}(t)$. The total number of elements of $T_{h,c}$ will be denoted by *N*. The total number of vertices of $T_{h,c}$ will be denoted by N and the number of vertices on the boundary as $N_{s}$. We define the Lagrangian finite element spaces on $T_{h,c}$ as

(16) $$\begin{array}{ccc} L^{1}(\Omega_{c,h}) & = & {\left\{{\overset{ˆ}{v}}_{h}\in H^{1}(\Omega_{c,h})\;:\;{\overset{ˆ}{v}}_{h}\right|}_{K}\in P_{1}(K),\;\;\forall \;K\in T_{h,c}\},\\ L_{0}^{1}(\Omega_{c,h}) & = & {\left\{{\overset{ˆ}{v}}_{h}\in H^{1}(\Omega_{c,h})\;:\;{\overset{ˆ}{v}}_{h}\right|}_{K}\in L^{1}(\Omega_{c,h})\;:\;{\overset{ˆ}{v}}_{h}=0,\;\;\xi \in \Gamma_{c,h}\}, \end{array}$$

where $P_{1}(K)$ is the space of linear polynomials on *K*.

In section 4 we will describe a procedure for evolving the nodal positions of the triangulation covering $\Omega_{h}(t)$. Given the location of the mesh nodes, the ALE mapping will be interpolated using piecewise linear elements giving rise to a discrete mapping $A_{h,t}\in L^{1}{\left(\Omega_{c,h}\right)}^{2}$ of the form

(17) $$x_{h}(\xi ,t)=A_{h,t}(\xi )=\sum_{i=1}^{N}x_{i}(t){\overset{ˆ}{\phi }}_{i}(\xi ),$$

where $x_{i}(t)=A_{h,t}(\xi_{i})$ denotes the position of node *i* at time *t*, and ${\overset{ˆ}{\phi }}_{i}$ is the associated nodal basis function in $L^{1}(\Omega_{c,h})$. The discretised ALE velocity therefore takes the form

$$w_{h}(\xi ,t)=\sum_{i=1}^{N}{\overset{˙}{x}}_{i}(t){\overset{ˆ}{\phi }}_{i}(\xi ).$$

Let $T_{h,t}$ be the image of the reference triangulation $T_{h,c}$ under the discrete ALE mapping $A_{h,t}$. Since the mapping is linear, each $K_{t}$ which is the image of a triangle $K\in T_{h,c}$, is also a triangle with straight edges. Using the ALE mapping, the finite element test space on $\Omega_{h}(t)$ is therefore defined as

$$H_{h}(\Omega_{h}(t))=\{v_{h}\;:\;\Omega_{h}(t)\to R\;:\;v_{h}={\overset{ˆ}{v}}_{h}\circ A_{h,t}^{-1},\;\overset{ˆ}{v}\in L^{1}(\Omega_{c,h})\}.$$

The finite element spatial discretisation of the conservative ALE formulation (14) therefore takes the form: find $c_{h}(t)\in H_{h}(\Omega_{h}(t))$ such that

(18) $$\begin{array}{c} \frac{d}{dt}\int_{\Omega_{h}(t)}c_{h}v_{h}\;dx-\int_{\Omega_{h}(t)}(\nabla \cdot (w_{h}c_{h}))v_{h}\;dx+D\int_{\Omega_{h}(t)}\nabla c_{h}\cdot \nabla v_{h}\;dx+\int_{\Gamma_{h}(t)}(g-(u\cdot n)c_{h})v_{h}\;ds\\ \;=\int_{\Omega_{h}(t)}fv_{h}\;dx,\;\;\forall v_{h}\in H_{h}(\Omega_{h}(t)). \end{array}$$

Similarly, on the boundary, we have the weak formulation: find $c_{s,h}\in H_{s,h}(\Gamma_{h}(t))$ such that

(19) $$\begin{array}{c} \frac{d}{dt}\int_{\Gamma_{h}(t)}c_{s,h}v_{s,h}\;ds+\int_{\Gamma_{h}(t)}(\nabla_{\Gamma }\cdot ((u-w_{h})c_{s,h}))\;v_{s,h}\;ds+D_{s}\int_{\Gamma_{h}(t)}\nabla_{\Gamma }c_{s,h}\cdot \nabla_{\Gamma_{h}}v_{s,h}\;ds\\ \;=\int_{\Gamma_{h}(t)}(g+h)v_{s,h}\;ds,\;\forall v_{s,h}\in H_{s,h}(\Gamma_{h}(t)). \end{array}$$

The finite element approximation of the bulk and surface species can be expressed as

$$c_{h}(x,t)=\sum_{j=1}^{N}c_{j}(t)\phi_{j}(x,t),\;\text{and}\;c_{s,h}(x,t)=\sum_{j=1}^{N_{s}}c_{s,j}(t)\phi_{s,j}(x,t),$$

where ${\left\{\phi_{j}(x,t)\right\}}_{j=1}^{N}$ and ${\left\{\phi_{s,j}(x,t)\right\}}_{j=1}^{N_{s}}$ are the time-dependent bulk and surface nodal basis functions. If $C(t)={\left\{c_{j}(t)\right\}}_{j=1}^{N}$ and $C_{s}(t)={\left\{c_{s,j}(t)\right\}}_{j=1}^{N_{s}}$, we can express (18) as the system of ordinary differential equations

(20) $$\frac{d}{dt}(M(t)C(t))+[K(t)+A(t,w_{h}(t))-B(t,w_{h}(t))]C(t)+D(C(t),C_{s}(t))=F(C(t)),$$

where

$${\left[M(t)\right]}_{ij}=\int_{\Omega_{h}(t)}\phi_{i}(t)\phi_{j}(t)\;dx$$

is the (time-dependent) mass matrix, while

$${\left[K(t)\right]}_{ij}=D\int_{\Omega_{h}(t)}(\nabla \phi_{j}(t)\cdot \nabla \phi_{i}(t))\;dx,$$

$${\left[A(t,w_{h}(t))\right]}_{ij}=\int_{\Gamma_{h}(t)}[(u-w_{h})\cdot n]\phi_{i}(t)\phi_{j}(t)\;ds,$$

$${\left[B(t,w_{h}(t))\right]}_{ij}=-\int_{\Omega_{h}(t)}[w_{h}\cdot \nabla \phi_{i}(t)]\phi_{j}(t)\;dx,$$

$${\left[D(C(t),C_{s}(t))\right]}_{i}=\int_{\Gamma_{h}(t)}[g(c_{h}(t),c_{s,h}(t))-(u\cdot n)c_{h}(t)]\phi_{i}(t)\;ds,$$

and the load vector

$${\left[F(C(t))\right]}_{i}=\int_{\Omega_{h}(t)}f(c_{h}(t))\phi_{i}(t)\;dx.$$

Note that the vector ***D*** will be sparse as only those values of *i* corresponding to boundary vertices will be non-zero. The spatial discretisation of the boundary equation (19) results in a system of ODEs

(21) $$\frac{d}{dt}(M_{s}(t)C_{s}(t))+[K_{s}(t)+A_{s}(t,w_{h}(t))]C_{s}(t)=D_{s}(C(t),C_{s}(t))+H(C_{s}(t)),$$

where

$${\left[M_{s}(t)\right]}_{ij}=\int_{\Gamma_{h}(t)}\phi_{s,i}(t)\phi_{s,j}(t)\;ds,$$

$${\left[K_{s}(t)\right]}_{ij}=D_{s}\int_{\Gamma (t)}(\nabla_{\Gamma }\phi_{s,j}(t)\cdot \nabla_{\Gamma }\phi_{s,i}(t))\;ds,$$

$${\left[A_{s}(t,w_{h}(t))\right]}_{ij}=\int_{\Gamma_{h}(t)}\left([\nabla_{\Gamma }\cdot (u-w_{h})]\phi_{s,i}(t)\phi_{s,j}(t)+[(u-w_{h})\cdot \nabla_{\Gamma }\phi_{s,j}(t)]\phi_{s,i}(t)\right)\;ds,$$

$${\left[H(C_{s}(t))\right]}_{i}=\int_{\Gamma_{h}(t)}h(c_{s,h}(t))\phi_{s,i}(t)\;ds,$$

and $D_{s}$ are the appropriately reordered non-zero elements of ***D***.

### 3.2 Temporal integration

To obtain a temporal discretisation of (20) and (21) we subdivide [0,T] into $N_{t}$ equal time intervals of size $\Delta t=T/N_{t}$ and denote $t^{n}=n\Delta t$, $n=0,1,\ldots ,N_{t}$. We will discretise the ALE mapping using linear interpolation between time levels. That is we will define

(22) $$A_{h,\Delta t}(\xi ,t)=\frac{t-t^{n}}{\Delta t}A_{h,t^{n+1}}(\xi )+\frac{t^{n+1}-t}{\Delta t}A_{h,t^{n}}(\xi ),\;t\in [t_{n},t_{n+1}),$$

where $A_{h,t}$ is the piecewise linear map at time *t*. The mesh velocity is therefore piecewise constant in time and is given by

(23) $$w_{h,\Delta t}^{n+1}(\xi )=\frac{A_{h,t^{n+1}}-A_{h,t^{n}}}{\Delta t},\;t\in [t^{n},t^{n+1}),$$

$$w_{h,\Delta t}^{n+1}(x,t)=w_{h,\Delta t}^{n+1}(\xi )\circ A_{h,\Delta t}^{-1}(x).$$

The temporal discretisation of the coupled systems (20) and (21) is obtained using a modified Crank–Nicolson semi-implicit approach. We first predict the boundary solution ${\overset{˜}{C}}_{s}^{n+1}$ using a semi-implicit backward Euler method, where the linear diffusion and mesh movement terms are treated implicitly, and the nonlinear reaction and coupling terms are treated explicitly. The predicted boundary solution therefore satisfies the linear system

(24) $$[M_{s}^{n+1}+\Delta t(K_{s}^{n+1}+A_{s}^{n+1})]{\overset{˜}{C}}_{s}^{n+1}=M_{s}^{n}C_{s}^{n}+\Delta t[D_{s}(C^{n},C_{s}^{n})+H(C_{s}^{n})].$$

The bulk approximation is then updated using a Crank–Nicolson step

(25) $$\begin{array}{c} {}[M^{n+1}+\frac{1}{2}\Delta t(K^{n+1}+A^{n+1}+B^{n+1})]C^{n+1}\\ \;=[M^{n}-\frac{1}{2}\Delta t(K^{n}+A^{n}+B^{n})]C^{n}+\frac{1}{2}\Delta t[F(C^{n+1})+F(C^{n})-D(C^{n+1},{\overset{˜}{C}}_{s}^{n+1})-D(C^{n},C_{s}^{n})]. \end{array}$$

The nonlinear system (25) is solved using Newton iteration. Finally, to ensure that the boundary solution is second-order in time, we perform a Crank–Nicolson correction step

(26) $$\begin{array}{c} {}[M_{s}^{n+1}+\frac{1}{2}\Delta t(K_{s}^{n+1}+A_{s}^{n+1})]C_{s}^{n+1}\\ \;=[M_{s}^{n}-\frac{1}{2}\Delta t(K_{s}^{n}+A_{s}^{n})]C_{s}^{n}+\frac{1}{2}\Delta t[D_{s}(C^{n+1},{\overset{˜}{C}}_{s}^{n+1})+D_{s}(C^{n},C_{s}^{n})+H({\overset{˜}{C}}_{s}^{n+1})+H(C_{s}^{n})]. \end{array}$$

Note that this correction step only requires the solution of a linear system of equations even if the reaction terms on the surface, *g* and *h* are nonlinear. The linear systems arising above are solved using the iterative method BiCGSTAB [52] and an incomplete LU (ILU) factorisation as a preconditioner.

For the cell migration application considered later, we note that the diffusive time scales in the extracellular region are often much shorter than the time scale associated with cell migration. As the time integration scheme above is fully implicit in the diffusive terms it is therefore robust to the choice of the time step for these applications.

### 3.3 A model bulk–surface problem on a stationary domain

To get an indication of the spatial and temporal convergence rate of the coupled bulk–surface finite element discretisation, we apply it to the solution of the following model problem:

(27) $$\frac{\partial c}{\partial t}=\Delta c,\;\;x\in \Omega$$

(28) $$\frac{\partial c_{s}}{\partial t}=\Delta_{\Gamma }c_{s}+c-c_{s},\;x\in \Gamma ,$$

(29) $$-\frac{\partial c}{\partial n}=c-c_{s},\;x\in \Gamma ,$$

where Ω is the unit circle.

This problem can be tackled analytically using polar coordinates x=rcos⁡θ, y=rsin⁡θ so that

$$\frac{\partial c}{\partial t}=\frac{\partial^{2}c}{\partial r^{2}}+\frac{1}{r}\frac{\partial c}{\partial r}+\frac{1}{r^{2}}\frac{\partial^{2}c}{\partial \theta^{2}},\;\;x\in \Omega$$

$${\frac{\partial c_{s}}{\partial t}=\frac{\partial^{2}c_{s}}{\partial \theta^{2}}+c|}_{r=1}-c_{s},\;x\in \Gamma ,$$

and

$${{-\frac{\partial c}{\partial r}|}_{r=1}=c|}_{r=1}-c_{s}.$$

Similar to Novak et al. [43], we look for a solution in the form

$$c_{s}(\theta ,t)=Ae^{-k^{2}t}\cos\theta ,$$

and

$$c(r,\theta ,t)=\rho (r)c_{s}(\theta ,t).$$

The following exact solution has been used to test the convergence of the finite element discretisation:

$$c(r,\theta ,t)=J_{1}(rk)e^{-k^{2}t}\cos\theta$$

and

$$c_{s}(\theta ,t)=\frac{J_{1}(k)}{2-k^{2}}e^{-k^{2}t}\cos\theta ,$$

where $J_{1}$ is the first-order Bessel function of the first kind and k=1.177706027.

**Fig. 2:**
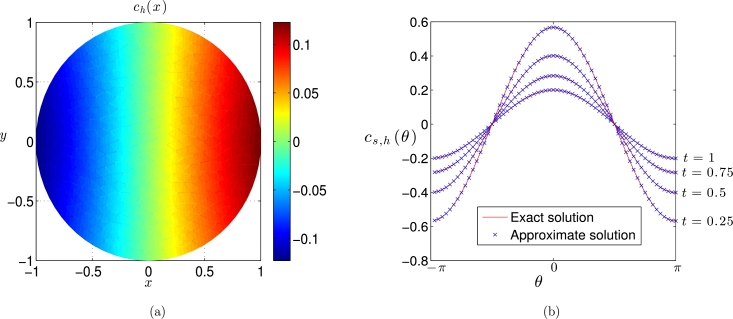
Numerical solution of coupled model problem (27), (28), (29) on a stationary unit circle. (a) Approximate bulk solution *c*_*h*_(***x***) at *t* = 1. (b) Approximate solution *c*_*s*,*h*_(*θ*) on the boundary compared to the exact solution.

**Fig. 3:**
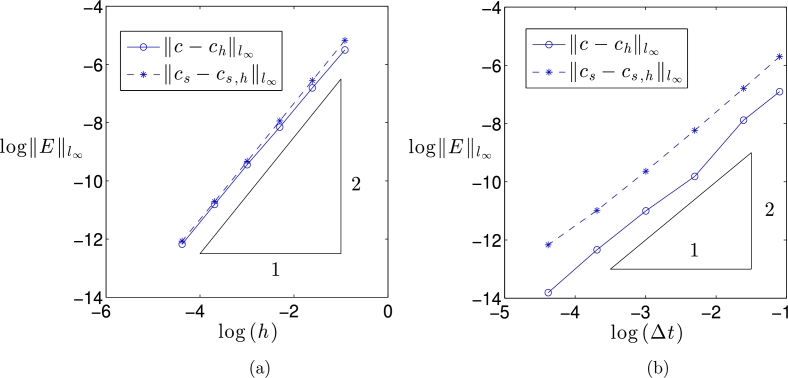
Second-order convergence of the maximum nodal error as (a) *h* → 0 and (b) Δ*t* → 0 for a coupled model problem on a unit circle.

Fig. 2 shows the computed approximate solutions in the bulk domain and on the domain boundary using an isotropic mesh with maximal cell diameter h=0.1 and a time step $\Delta t=2\times 10^{-4}$. We can see that the method performs well for these values of the discretisation parameters. To test the spatial rate of convergence of the algorithm, simulations were performed on a sequence of increasingly refined isotropic meshes. To ensure that the error was dominated by its spatial component, a sufficiently small time step of $\Delta t=10^{-3}$ was used. Fig. 3(a) shows the maximum error over all grid nodes for both the bulk and surface numerical solutions and we can see that both converge at the rate of $O(h^{2})$, as expected. To investigate the temporal rate of converge, simulations were performed using a fine mesh with N=150,000 elements and various time steps. We can see from Fig. 3(b), that the three-step solution procedure (24), (25), (26) furnishes approximations which are second-order accurate in time. Note that if the surface solution correction step (26) were omitted then, as expected, the resulting approximations were only temporally first-order accurate.

## 4 Practical evaluation of ALE mapping

For the ALE mapping to be useful, it must satisfy a number of properties. First, it must be inexpensive to construct, relative to the cost of solving the physical problem. Second, the resulting meshes covering Ω(t) must be of good quality in the sense that mesh elements are adapted to salient features such at steep boundary or interior layers in the physical solution. Finally, the meshes must evolve smoothly in time to avoid the need to use small time steps to maintain numerical stability.

### 4.1 Bulk domain mesh generation

**Fig. 4:**
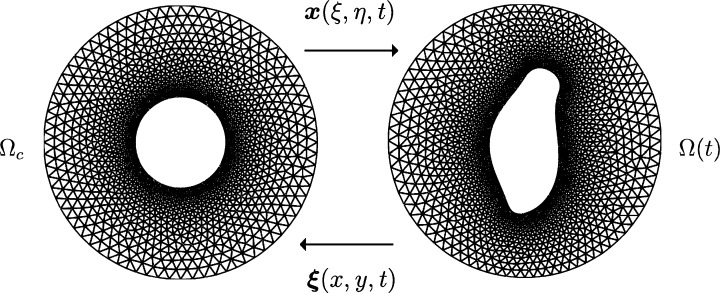
A moving mesh covering Ω(*t*) is the image of a fixed mesh on a reference domain Ω_*c*_ through a time-dependent ALE mapping $A_{t}(\xi )=x(\xi ,t)$.

To avoid potential mesh crossings or foldings, we derive a suitable evolution equation for the inverse ALE mapping $A_{t}^{-1}(x)=\xi (x,t)$ rather than $A_{t}(\xi )=x(\xi ,t)$ (see, for example, the discussion in [12]). As shown in Fig. 4, a mesh $T_{h,t}$ on $\Omega_{h}(t)$ can then be generated as the preimage of a fixed mesh $T_{h,c}$ on $\Omega_{c,h}$. As introduced in [22], we choose the mapping ξ(x) corresponding to a fixed value of *t* in order to minimise the functional

(30) $$I[\xi ]=\frac{1}{2}\int_{\Omega_{t}}[{\left(\nabla \xi \right)}^{T}G^{-1}(\nabla \xi )+{\left(\nabla \eta \right)}^{T}G^{-1}(\nabla \eta )]\;dx,$$

where *G* is a 2×2 symmetric positive definite matrix referred to as a monitor matrix and ∇ is the gradient operator with respect to ***x***. It can be shown that the functional (30) is coercive and uniformly convex and hence has a unique minimiser [23].

Rather than directly attempt to minimise (30), a more robust procedure is to evolve the mapping according to the modified gradient flow equations

(31) $$\frac{\partial \xi }{\partial t}=\frac{P}{\tau }\nabla \cdot (G^{-1}\nabla \xi ),\;\;\text{and }\;\;\frac{\partial \eta }{\partial t}=\frac{P}{\tau }\nabla \cdot (G^{-1}\nabla \eta ).$$

Here, τ>0 is a user-specified temporal smoothing parameter which affects the temporal scale over which the mesh adapts and *P* is a positive function of (x,t), chosen such that the mesh movement has a spatially uniform time scale [19]. The gradient flow structure of (31) ensures that the mesh evolves smoothly in time and improves robustness in the choice of the initial mesh.

The selection of an appropriate monitor matrix is crucial to the success of mesh adaptation. In this paper, we will consider the monitor matrix proposed by Winslow [53]

(32) $$G=\left[\begin{array}{cc} M\; & 0\\ 0\; & M \end{array}\right],$$

where M(x,t) is a positive weight function called a *monitor function*. A suitable monitor function is essential to the success of adaptive moving mesh methods. The monitor function should be taylored to the type of PDE being solved and the numerical method being used. Monitor functions based on interpolation estimates, a posteriori error estimates and adaptation to solution features have all been proposed [3], [8], [23]. If no such estimate exists then the monitor function could be any smooth function designed to adapt the mesh towards important solution features such moving interfaces or boundaries (see for example [5]).

In practice, we interchange the roles of the dependent and independent variables in (31), since it's the location of the physical mesh points ${\left\{x_{i}(t)\right\}}_{i=1}^{N}$ that defines the ALE map. With a Winslow-type monitor matrix (32) the resulting MMPDEs take the form

(33) $$\tau \frac{\partial x}{\partial t}=P(ax_{\xi \xi }+bx_{\xi \eta }+cx_{\eta \eta }+dx_{\xi }+ex_{\eta }),\;(\xi ,\eta )\in \Omega_{c},$$

where

$$a=\frac{1}{M}\frac{x_{\eta }^{2}+y_{\eta }^{2}}{J^{2}},\;b=-\frac{2}{M}\frac{\left(x_{\xi }x_{\eta }+y_{\xi }y_{\eta }\right)}{J^{2}},\;c=\frac{1}{M}\frac{x_{\xi }^{2}+y_{\xi }^{2}}{J^{2}},$$

$$d=\frac{1}{{\left(MJ\right)}^{2}}[M_{\xi }(x_{\eta }^{2}+y_{\eta }^{2})-M_{\eta }(x_{\xi }x_{\eta }+y_{\xi }y_{\eta })],\;e=\frac{1}{{\left(MJ\right)}^{2}}\left[-M_{\xi }(x_{\xi }x_{\eta }+y_{\xi }y_{\eta })+M_{\eta }(x_{\xi }^{2}+y_{\xi }^{2})\right],$$

and $J=x_{\xi }y_{\eta }-x_{\eta }y_{\xi }$ is the Jacobian of the ALE mapping. To complete the specification of the coordinate transformation, the MMPDE must be supplemented by initial and boundary conditions. In all the applications described later, the computational domain $\Omega_{c,h}=\Omega_{h}(0)$, and the initial mesh over the physical domain is obtained using the Matlab toolbox Distmesh [46]. To allow the mesh points to move along the boundary $\Gamma_{h}(t)$, they are obtained using a one-dimensional moving mesh approach outlined in section 4.2. Once the boundary nodes have been evolved over one time step, they then become Dirichlet conditions completing the specification for (33).

The numerical solution of (33) requires spatial and temporal discretisation. In space, we discretise using standard linear Galerkin finite elements. In the time direction, we use a backward Euler integration scheme to update the solution at $t=t^{n+1}$, and to avoid solving nonlinear algebraic systems, we evaluate the coefficients a,c,…,e at the time $t=t^{n}$. We therefore seek $x_{h}^{n+1}\in {\left(L^{1}(\Omega_{c,h})\right)}^{2}$ such that

(34) $$\begin{array}{c} \tau \int_{\Omega_{c,h}}\left(\frac{x_{h}^{n+1}-x_{h}^{n}}{\Delta t}\right)\cdot {\overset{ˆ}{v}}_{h}\;d\xi +\int_{\Omega_{c,h}}[{\left(x_{h}^{n+1}\right)}_{\xi }\cdot {\left(a^{n}{\overset{ˆ}{v}}_{h}\right)}_{\xi }+{\left(x_{h}^{n+1}\right)}_{\eta }\cdot {\left(c^{n}{\overset{ˆ}{v}}_{h}\right)}_{\eta }\\ \;+\frac{1}{2}[{\left(x_{h}^{n+1}\right)}_{\xi }\cdot {\left(b^{n}{\overset{ˆ}{v}}_{h}\right)}_{\eta }+{\left(x_{h}^{n+1}\right)}_{\eta }\cdot {\left(b^{n}{\overset{ˆ}{v}}_{h}\right)}_{\xi }]-[d^{n}{\left(x_{h}^{n+1}\right)}_{\xi }+e^{n}{\left(x_{h}^{n+1}\right)}_{\eta }]\cdot {\overset{ˆ}{v}}_{h}]\;d\xi =0, \end{array}$$

for all ${\overset{ˆ}{v}}_{h}\in {\left(L_{0}^{1}(\Omega_{c,h})\right)}^{2}$. The resulting linear systems are again solved using the iterative method BiCGSTAB and an incomplete LU (ILU) factorisation as a preconditioner. An analysis of the performance of this iterative solver for the discretised MMPDE equations can be found in [4].

### 4.2 Boundary mesh generation

We consider the class of boundary movements given by the following evolution law for the normal velocity

(35) V(x,t)=ακ+β,x∈Γ(t),

where *κ* is the curvature. Within the context of biological cell applications, the functions *α* and *β* could model the physical forces exerted on the membrane by cortical tension, and protrusions caused by actin polymerisation, respectively.

We consider the solution of (35) using a Lagrangian approach, where the main idea is to represent the flow of curves by the position vector ***x*** which is the solution of the geometric evolution equation

(36) $$\overset{˙}{x}=Vn+Bt,$$

where ***n*** and ***t*** are unit normal and tangent vectors, respectively. Note that the presence of the tangential velocity B has no effect on the shape of the evolving curve but it is well known that the incorporation of a suitably chosen non-zero value of B can avoid the major drawback of the Lagrangian approach which is the possibility that grid points merge, resulting in a loss of stability and accuracy [2], [1], [37].

An embedded regular plane curve Γ can be parameterised by the smooth function $x\;:\;S^{1}\to R^{2}$, i.e. $\Gamma =\mathrm{Image}(x):=\{x(\sigma ),\;\sigma \in S^{1}\}$. Taking into account the periodic boundary conditions at σ=0,1, we shall hereafter identify $S^{1}$ with the interval [0,1]. The unit arc-length parameterisation will be denoted by *s* and it can be shown that $x_{s}=t$ and $x_{ss}=\kappa n$. The arc-length of the curve from the point $x(\sigma_{0})$ to x(σ) is given by

$$s(\sigma )=\int_{\sigma_{0}}^{\sigma }\sqrt{x_{\sigma }^{2}+y_{\sigma }^{2}}\;d\sigma =\int_{\sigma_{0}}^{\sigma }\|x_{\sigma }\|\;d\sigma ,$$

where ‖⋅‖ denotes the $l_{2}$-norm. Using the chain rule

(37) $$x_{\sigma }=x_{s}\frac{ds}{d\sigma }=x_{s}\|x_{\sigma }\|$$

and differentiation of (37) leads to the relation

(38) $$x_{\sigma \sigma }=x_{ss}{\left\|x_{\sigma }\right\|}^{2}+x_{s}{\left\|x_{\sigma }\right\|}_{s}\|x_{\sigma }\|.$$

Multiplying through (38) by ***n***, we get an expression for *κ* and hence in terms of the parameterisation *σ*, we can express the normal velocity as

(39) $$V=\overset{˙}{x}\cdot n=\alpha \left(\frac{x_{\sigma \sigma }\cdot n}{{\left\|x_{\sigma }\right\|}^{2}}\right)+\beta .$$

Control of the mesh spacing in the tangential direction can be achieved by ensuring that a weighted arc-length between grid nodes is constant. That is, if M(x,t)>0 is a positive monitor function, then we set

$$M\;\frac{ds}{d\sigma }=\text{constant}$$

and hence

(40) $${\left(M\;\frac{ds}{d\sigma }\right)}_{\sigma }=(M\|x_{\sigma }{\left\|\right)}_{\sigma }=0.$$

The differential-algebraic system (39) and (40) could, in theory, be used to evolve the boundary Γ(t); the case α=1, β=0 and M=1, which aims to construct a uniform arc-length parameterisation, is exactly the equation system used by Pan and Wetton [45]. In [39] we used the parameterised finite element method (PFEM) [2] to evolve the approximation of a curve describing an evolving cell membrane. This method introduces an intrinsic tangential velocity B such that the arc-length between grid nodes is equidistributed. When using the PFEM to generate boundary node displacements, which are then used as boundary data for meshes covering bulk regions, we have found that the lack of control of the PFEM intrinsic tangential velocity can lead to poor quality bulk meshes which require frequent remeshing. Experience with the generation of adaptive moving meshes suggests however that the introduction of an evolution equation for the tangential velocity, driven by the mesh equidistribution condition (40), can significantly improve stability and robustness [21], [20], [3]. Therefore, we consider the tangential velocity equation

(41) $$B=\overset{˙}{x}\cdot t=\frac{P}{\tau }(M\|x_{\sigma }{\left\|\right)}_{\sigma },$$

where, as before, *τ* and *P* are a temporal smoothing parameter and spatial balancing operator, respectively. The proposed procedure therefore involves the simultaneous solution of (39) and (41).

For simplicity, we discretise (39) and (41) using a finite difference method. The evolving curve at time $t^{n}$ is represented by the discrete plane points $x_{i}^{n}$, $i=1,\ldots ,N_{s}$. The linear approximation of the curve is therefore given by the polygon with vertices $x_{i}^{n}$, $i=1,\ldots ,N_{s}$. Due to the periodicity conditions, we also use the additional values $x_{-1}^{n}=x_{N_{s}-1}^{n}$, $x_{0}^{n}=x_{N_{s}}^{n}$ and $x_{N_{s}+1}^{n}=x_{1}^{n}$. We use a uniform discretisation of the parameterisation interval σ∈[0,1] with step size $\Delta \sigma =1/N_{s}$. We use central finite differences to approximate the spatial derivatives in (39) and a first-order fully implicit temporal discretisation. This leads to a nonlinear system which is solved using Picard iteration. Let $x^{\left[n,m\right]}$ denote the approximation of ***x*** at time level $t^{n}$ and iteration level *m*, then for the normal velocity equations we have the linear system

(42) $$\begin{array}{c} {}[-\mu_{i}^{\left[n+1,m\right]}x_{i-1}^{\left[n+1,m+1\right]}+(1+2\mu_{i}^{\left[n+1,m\right]})x_{i}^{\left[n+1,m+1\right]}-\mu_{i}^{\left[n+1,m\right]}x_{i+1}^{\left[n+1,m+1\right]}]\cdot n_{i}^{\left[n+1,m\right]}\\ \;=x_{i}^{\left[n+1,m\right]}\cdot n_{i}^{\left[n+1,m\right]}+\Delta t\beta_{i}, \end{array}$$

for $i=1,\ldots ,N_{s}$, where $\mu_{i}^{\left[n,m\right]}=4\Delta t\alpha_{i}/({\left\|x_{i+1}^{\left[n,m\right]}-x_{i-1}^{\left[n,m\right]}\right\|}^{2})$. We approximate the unit tangent vector

$$t_{i}^{n}=\frac{x_{i+1}^{n}-x_{i-1}^{n}}{\left\|x_{i+1}^{n}-x_{i-1}^{n}\right\|}=(t_{1}^{n},t_{2}^{n}),$$

and hence we set $n_{i}^{n}=(t_{2}^{n},-t_{1}^{n})$. Using a similar discretisation of the tangential velocity equation (41) we have

(43) $$\begin{array}{c} {}[-\nu_{i}^{n}x_{i-1}^{\left[n+1,m+1\right]}+(1+2\nu_{i}^{n})x_{i}^{\left[n+1,m+1\right]}-\nu_{i}^{n}x_{i+1}^{\left[n+1,m+1\right]}]\cdot t_{i}^{\left[n+1,m\right]}\\ \;=x_{i}^{n}\cdot t_{i}^{\left[n+1,m\right]}+\frac{\Delta tP_{i}}{4\tau {\left(\Delta \sigma \right)}^{2}}(M_{i+1}^{n}-M_{i-1}^{n})(\|x_{i+1}^{\left[n+1,m\right]}-x_{i-1}^{\left[n+1,m\right]}\|), \end{array}$$

for $i=1,\ldots ,N_{s}$, where $\nu_{i}^{n}=\Delta tM_{i}^{n}P_{i}/(\tau {\left(\Delta \sigma \right)}^{2})$. The coupled set of $2N_{s}$ equations (42) and (43) are solved for $x^{\left[n+1,m+1\right]}$ and the Picard iteration is stopped when

$$\|x^{\left[n+1,m+1\right]}-x^{\left[n+1,m\right]}\|<\text{TOL}.$$

The last iteration is then used as the approximation $x^{n+1}$.

### 4.3 Example

To demonstrate the capability of the proposed adaptive grid generation procedures, we consider the evolution of an ellipse under mean curvature flow where V=−ακ, and simultaneously we require the bulk and surface meshes evolve to resolve the travelling wave profile

$$u(x,y,t)=\frac{1}{2}\left[1+\tanh\left(\frac{x+t-0.7}{0.6}\right)\right].$$

There are many possible choices for the monitor function but for illustrative purposes we will set

$$M(x,y,t)=1+{\mathrm{sech}}^{2}\left(\frac{x+t-0.7}{0.6}\right).$$

**Fig. 5:**
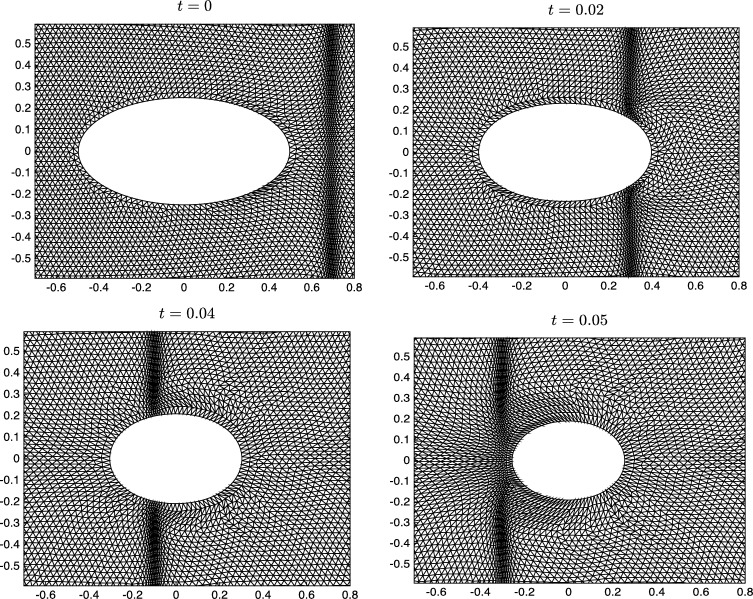
Adaptive bulk meshes for a time-dependent domain where the inner boundary evolves by mean curvature flow. The meshes are adapted to a travelling wave profile moving across the domain from right to left.

**Fig. 6:**
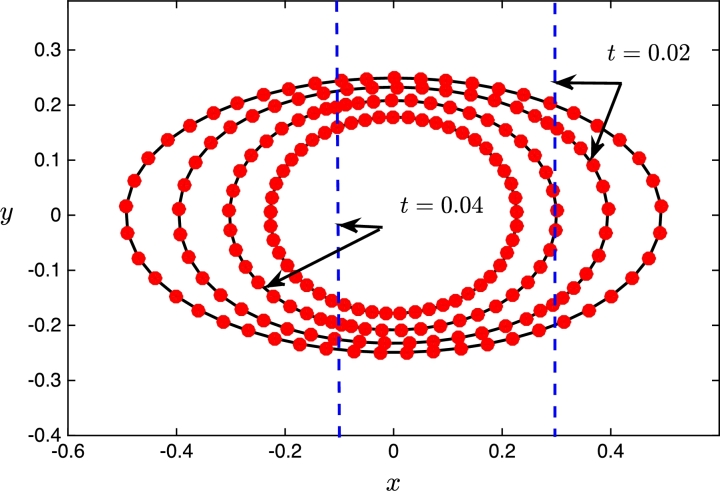
Computed meshes for a curve evolving in the normal direction by mean curvature flow; the meshes are also adapted in the tangential direction to a travelling wave profile moving across the domain from right to left. Meshes are shown at *t* = 0, 0.02, 0.04 and *t* = 0.05. The dashed vertical lines correspond to the location of the wave front at the times indicated.

**Fig. 7:**
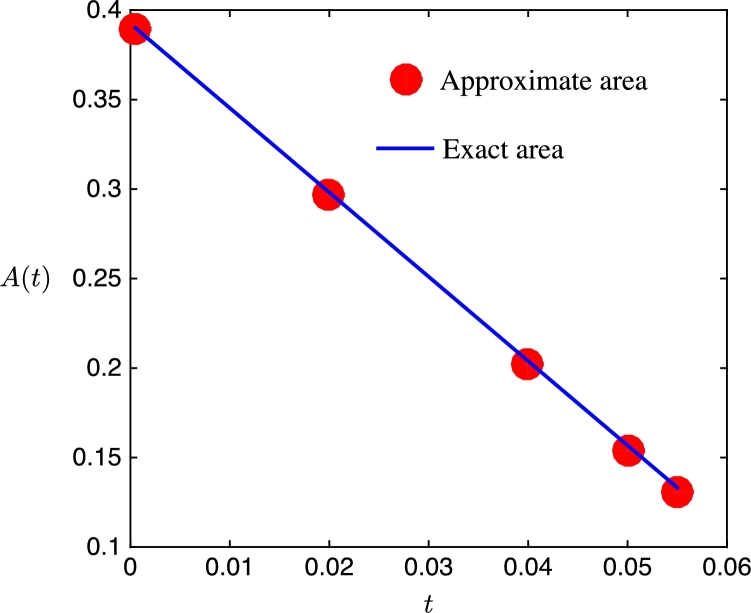
Comparison of the evolution of the exact and computed area enclosed by a closed curve under mean curvature flow at *t* = 0, 0.02, 0.04, 0.05 and *t* = 0.055.

The monitor function takes its maximum value at the centre of the travelling wave and decreases smoothly to a non-zero constant value far from the front. The resulting adapted mesh should therefore be clustered around the wave but almost uniform in the flat regions. The initial physical domain consists of the region exterior to the ellipse $4x^{2}+16y^{2}=1$ and interior to a unit circular far-field boundary; the fixed computational domain $\Omega_{c}$ is chosen to be the initial physical domain. The computational mesh is obtained using the Matlab toolbox Distmesh [46] and has N=9832 elements and $N_{s}=98$ vertices on the boundary of the ellipse. The simulations are performed over the time interval $t\in [0,7\times 10^{-2}]$ using a constant time step $\Delta t=10^{-3}$. The coefficient of the normal velocity of the evolving curve is α=0.75, and the temporal smoothing parameters in the moving mesh PDEs are both $\tau =10^{-4}$. The computed meshes at four representative times are shown in Fig. 5. We can see that the initial mesh is adapted towards the wave front which is centred to the right of the ellipse at x=0.7. Initially the boundary mesh points are distributed to equidistribute the arc-length as the monitor function M≈1 along the ellipse. At t=0.02 and t=0.04, we can see that the bulk meshes have moved to follow the wave front; the boundary meshes have also adapted correctly in the tangential direction and evolved in the normal direction according to the curvature of the boundary. Finally, at t=0.05, the wave front has passed by the inner curve and the boundary mesh relaxes back to equidistribute the arc-length between mesh points. Fig. 6 shows the adapted boundary meshes; for clarity, at the two times when the wave front intersects the boundary, we have indicated its location by a vertical dashed line. We can see that at these times the boundary mesh has adapted well to the wave front and at the other two times the mesh equidistributes the arc-length of the evolving curve. To test the accuracy of the computed moving boundary, Fig. 7 shows the evolution of the exact and approximate area enclosed by the closed curve; the exact area for mean curvature flow takes the form A(t)=A(0)−2παt. We can see that the approximate area decreases linearly in time, and at the times indicated it agrees well with the exact area.

## 5 The complete algorithm

We now describe the complete algorithm implementing the ALE–FEM scheme and the generation of the surface and bulk meshes. At time $t=t^{n}$ we assume we have a mesh $T_{h,t^{n}}$ and the finite element approximations $c_{h}^{n}$ and $c_{s,h}^{n}$ of the bulk and surface species, respectively. The following steps are then carried out to advance the mesh and the finite element approximations.

1. *Update the physical mesh*
  (a) Using $c_{s,h}^{n}$, calculate the normal velocity of Γ given by (35).
  (b) Update the location of the manifold $\Gamma_{h}^{n+1}$ by solving the simultaneous systems (42) and (43).
  (c) Using the bulk approximation $c_{h}^{n}$, determine the mesh adaptation monitor function *M*.
  (d) Using the updated boundary points as fixed Dirichlet data, update the interior mesh points by solving (34).
  (e) Test for mesh quality. If the mesh is fine then goto 2. If not then re-grid and interpolate the solutions onto the new mesh and re-do the time step.
2. *Update the finite element solutions in the bulk and the surface*
  (a) Use the meshes $T_{h,t^{n+1}}$ and $T_{h,t^{n}}$ to define the discrete ALE velocity $w_{h}$.
  (b) Update the solutions $c_{s,h}^{n+1}$ and $c_{h}^{n+1}$ by solving (24), (25), (26).

To determine the quality of the bulk meshes we measure the minimum angle over all of the triangles and decide to remesh when this is below a given tolerance. The new mesh covering the physical domain is then used as the fixed computational mesh. The bulk and boundary solutions are then linearly interpolated onto the new mesh to allow further time integration.

## 6 Cell migration and chemotaxis

We now consider the application of the developed algorithm to the computational modelling of eukaryotic cell migration and chemotaxis. In [40], [41] we developed a “pseudopod-centered” [25] model based on a system of reaction–diffusion equations that gives rise to a suitable spatiotemporal activator profile that can be used for the generation of pseudopods without the need for a driving external signal. The following set of equations was derived from a well-established discrete model developed by Meinhardt [35] (M model). The model describes the dynamic interaction between a membrane-bound local autocatalytic activator *a*, a rapidly distributed global inhibitor *b* and a local inhibitor *c*. Assuming that the cell boundary Γ(t) moves with velocity ***u***, then for x∈Γ(t) the equations take the form

(44) $$\frac{\partial a}{\partial t}+\nabla_{\Gamma }\cdot (ua)=D_{a}\Delta_{\Gamma }a+\frac{s(a^{2}/b+b_{a})}{\left(s_{c}+c)(1+s_{a}a^{2}\right)}-r_{a}a,$$

(45) $$\frac{\partial b}{\partial t}+\nabla_{\Gamma }\cdot (ub)=D_{b}\Delta_{\Gamma }b-r_{b}b+\frac{r_{b}}{\left|\Gamma (t)\right|}\oint_{\Gamma (t)}a\;dx,$$

(46) $$\frac{\partial c}{\partial t}+\nabla_{\Gamma }\cdot (uc)=D_{c}\Delta_{\Gamma }c+b_{c}a-r_{c}c.$$

Here, $r_{a},r_{b}$ and $r_{c}$ denote decay rates of the local activator, global inhibitor and local inhibitor, respectively. The corresponding diffusion coefficients are $D_{a},D_{b}$ and $D_{c}$. In the nonlinear reaction term in the activator equation, $s_{a}$ is a saturation coefficient, $s_{c}$ is a Michaelis–Menten constant and $b_{a}$ is a basal production rate of the activator. The constant $b_{c}$ determines the growth of the local inhibitor *c* in the presence of the activator *a*. The effect of any external chemotactic field is incorporated in the signal term *s*. All of the variables appearing in this model are assumed to be non-dimensional. The activators and inhibitors in this model are not intended to represent molecular species. Although some molecular-based models have been successful in describing individual processes, the global morphology of real cells has proven too complex for such a description at present. We therefore have used a top-down approach, where each parameter can represent several molecular species. The pseudopod activator could read out at the level of actin nucleation, for example, through SCAR/WAVE proteins.

We assume that actin polymerisation creates a protrusive pressure that pushes the cell membrane outward in the normal direction. Recent detailed investigation of pseudopod formation suggests that this assumption is valid for cells migrating in the absence of external cues and in the presence of chemotactic gradients [5], [4]. We will assume that the rate of polymerisation is proportional to the concentration of the local activator. At rest, the cell experiences pressure from cortical tension, which maintains the spherical shape of the cell. Using a cortical shell-liquid drop model [7], the pressure generated by the cortical tension is assumed to act normally on the cell membrane and depends on the local surface curvature. The cell boundary is therefore assumed to evolve with the normal velocity

(47) $$V(x,t)=K_{\mathrm{prot}}a(x,t)-\lambda (t)\kappa ,\;x\in \Gamma (t).$$

To control the area enclosed by Γ(t), we have used a spatially constant but time dependent cortical tension factor λ(t). Larger values of *λ* will increase the cortical tension, which will eventually result in a decrease in the cell area. Conversely, by decreasing *λ* the cortical tension is weakened, leading to an increase in cell area. There are many possible forms that a dynamic equation for *λ* could take, but we have found through numerical experimentation that the following works well:

(48) $$\frac{d\lambda }{dt}=\frac{\lambda_{0}\lambda (A-A_{0}+dA/dt)}{A_{0}(\lambda +\lambda_{0})}-\beta \lambda .$$

Here, $\lambda_{0}$ and *β* are positive parameters and $A_{0}$ is the initial prescribed area of the cell. Equation (48) is solved numerically using an explicit Euler method.

The simulations of cell chemotaxis presented in [40], [41] were obtained using an approximation of the local fractional receptor occupancy, i.e. the ratio of the local number of ligand-bound receptors to the total number of receptors. The receptor occupancy was estimated using a ligand–receptor binding model which assumed the extracellular ligand field was unaffected by the binding process and the fact that the cell is moving and constantly changing morphology. While the predictions in [40], [41] displayed many attributes of real cell chemotaxis, we now consider a model to account for the interaction of the cell on the external chemotactic field.

In the extracellular region, Ω(t), we assume that the concentration of ligand molecules evolves according to a linear diffusion equation. At the cell membrane, Γ(t), we assume that a chemoattractant ligand *L* binds reversibly to a receptor *R* to form a receptor–ligand complex *LR*. The coupled bulk–surface system for the evolution of the ligand concentration, *l*, and the concentration of bound receptors, $l_{s}$, therefore takes the form

(49) $$\frac{\partial l}{\partial t}=D\Delta l,\;\;x\in \Omega (t)$$

(50) $$\frac{\partial l_{s}}{\partial t}+\nabla_{\Gamma }\cdot (ul_{s})=D_{s}\Delta_{\Gamma }l_{s}+k_{1}(R_{\mathrm{tot}}-l_{s})l-k_{-1}l_{s},\;\;x\in \Gamma (t).$$

Here, *D* and $D_{s}$ are the diffusion coefficients for the ligand and receptor–ligand complex, respectively. We assume that the total concentration of bound and unbound receptors is constant and takes the value $R_{\mathrm{tot}}$. The constant $k_{1}$ is the rate of ligand association and $k_{-1}$ the rate of disassociation. The normal flux boundary condition between the extracellular region and the cell membrane takes the form

(51) $$-D\frac{\partial l}{\partial n}-[(u\cdot n)]l=k_{1}(R_{\mathrm{tot}}-l_{s})l-k_{-1}l_{s},\;\;x\in \Gamma (t).$$

Determining the concentration of bound receptors, $l_{s}$ allows the estimation of the local fractional receptor occupancy

$$R_{o}(x,t)=\frac{l_{s}(x,t)}{R_{\mathrm{tot}}}.$$

It has been observed that some cells move randomly in the absence of any external cues. We have therefore included an intrinsic noise component to our system that is independent of the external chemotactic signal. For this purpose we will assume that the intrinsic noise $\eta^{t}$ satisfies a stochastic differential equation of mean reverting type [40]. The combined effect of the response to the external signal and random intrinsic noise is modelled by the term

$$s(x,t)=r_{a}(\eta^{t}+R_{o}(x,t)),$$

which feeds in multiplicatively to the autocatalytic activator equation (44).

### 6.1 Migration in a linear gradient

The following numerical experiments were performed using the parameters found in Table 1 and a uniform time step Δt=0.1. We first simulate a cell moving in a linear gradient of the chemoattractant field given initially by

(52) l(x,y)=5.3+8.5(x+0.1).

**Fig. 8:**
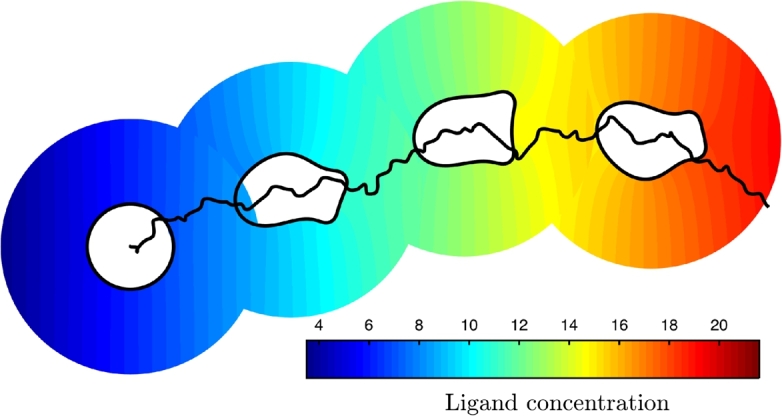
Simulation of single-cell chemotaxis. Although the cell's morphology is constantly changing by pseudopod extension and retraction, the cell's pathway exhibits a strong degree of directional persistence up the chemoattractant gradient to the right.

**Fig. 9:**
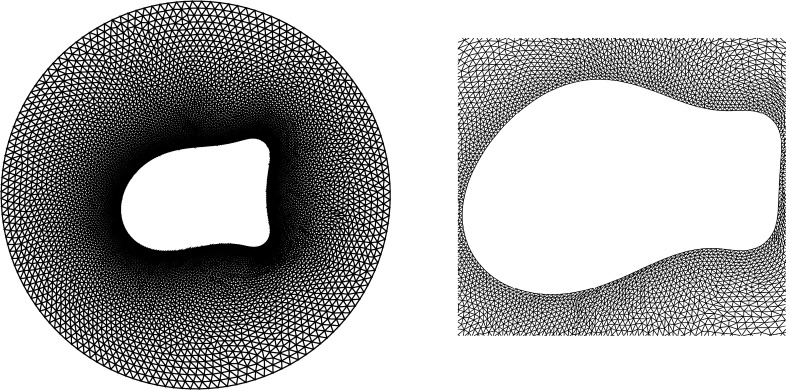
Global and close up view of a typical mesh used for the simulation of cell chemotaxis. The mesh follows faithfully the migrating cell and is of good quality close to the cell membrane.

With the receptor–ligand disassociation constant $K_{d}=k_{-1}/k_{1}=30$, the receptor occupancy initially at the back of the cell $R_{o}^{b}=0.15$, and at the front of the cell $R_{o}^{f}=0.19$. To improve computational efficiency when there is significant cell movement, the diffusion equation is solved over a time-dependent annular region Ω(t), where the internal boundary represents the cell membrane, and the outer far-field boundary is a circle centred on the moving cell. The radius of the far-field boundary $r_{f}=3r_{0}$, where the initial radius of the cell $r_{0}=0.1$. At the far-field boundary the ligand concentration is given by (52). The initial mesh has N=6250 elements and is adapted isotropically towards the cell membrane; this mesh is then kept fixed as the initial computational mesh covering $\Omega_{c}$. Fig. 8 shows four snap-shots of a simulated cell moving in the correct direction to the right. The cell migrates by the generation and splitting of pseudopods at the front of the cell while retracting the back. The generated mesh at one particular time is shown in Fig. 9. We can see that the bulk and boundary meshes are of high quality and follow the changing morphology of the migrating cell. Of the $5\times 10^{5}$ time steps used in this simulation, only 42 remeshing steps were required demonstrating the robustness of the grid generation algorithms. The computational method has been implemented in MATLAB to make use of the Distmesh algorithm for the generation of the initial mesh and meshes when re-gridding is needed. The simulations in this section took approximately 3 hours of computing time on a desktop machine using an Intel i7 2600 quad core processor running at 3.4 GHz. In this time the simulated cell had migrated a distance of approximately 10 cell diameters. No major attempt was made to optimise the code; it can be expected that run times would be reduced dramatically by computing in an appropriate low-level language.

### 6.2 Migration in an initially homogeneous field

**Fig. 10:**
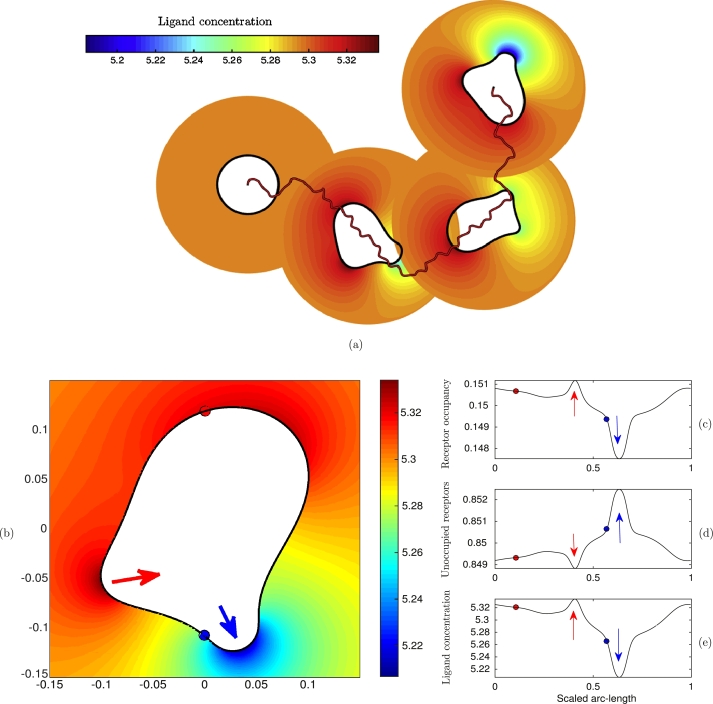
Simulation of cell migration in an initially homogeneous chemotactic field. (a) Cell outlines and ligand field at equal time intervals; (b) Close-up showing the generation of a pseudopod pushing outwards (blue arrow) and a retracting older pseudopod (red arrow); (c) Receptor occupancy on the cell membrane; (d) Proportion of the total number of receptors that are unoccupied and (e) the external ligand field at the cell membrane. (For interpretation of the references to colour in this figure legend, the reader is referred to the web version of this article.)

We now consider the possibility of a cell to shape an initially homogeneous ligand field and investigate the feedback of this interaction on the cell's migratory pattern. The biological interest in this problem comes from recent experimental observations that some migrating cells locally self-generate chemotactic gradients thus leading to increased persistence of migration or sustained directed migration [10], [38]. Fig. 10 shows a simulation of a typical cell, where the initial ligand concentration is constant throughout the extracellular domain. We can see that movement of the cell, and changes to the cell morphology affect the ligand field (a). In particular, when a new pseudopod is created (b), the cell pushes out its membrane and this results in the local dilution of the concentration of the receptor–ligand complex (c). As the total receptor concentration is assumed constant, this means that additional unbound receptors become available to bind ligand molecules at the cell membrane (d). The bulk concentration at the cell surface is then depleted resulting in a local gradient in the bulk ligand field towards the cell (e). Conversely, in areas where the cell membrane is retracted such as the back of the cell and at retracting pseudopods, the local concentration of receptor–ligand complex increases slightly and this creates a gradient in the bulk ligand field away from the cell surface which drives ligand molecules away from the cell surface in these areas. The overall effect of these interactions between the extracellular ligand field and the surface receptor binding kinetics is to enhance the polarity and the persistence of the migrating cell even though the unperturbed background field is homogeneous. Additional reactions such as the degradation of ligand molecules by membrane bound enzymes and receptor internalisation could also play a major role in the generation of self-generated gradients and we intend to consider these possibilities in the future.

## 7 Conclusions and further work

In this paper, we have developed a computational framework for the solution of coupled bulk–surface reaction–diffusion equations in two dimensions. The proposed algorithm is based on a conservative finite element ALE scheme to approximate the solution of the PDEs, and an adaptive moving mesh method for grid generation. A novel MMPDE approach has been developed to simulate a curve moving in the normal direction by a geometric evolution law that also allows control of the tangential distribution of mesh points. The overall algorithm has been shown to work well when applied to model problems with known analytical solutions. The method has also been applied to a model of cell migration and chemotaxis and shown to predict a possible novel mechanism for cells to increase their persistence and polarity by generating their own local gradients of chemoattractants. The numerical algorithm has proved to be very robust and requires few global remeshing steps even though the simulated cell morphology is constantly changing while it is migrating. The methodology is simple to implement using standard finite element procedures and freely available routines for initial grid generation.

In future, we aim to use the developed computational framework to tackle intracellular cell processes. The main issue there will be the proper description of the deformation of material points within the moving cell. This is of course a highly non-trivial issue although there have been attempts to model the cell mechanics as an active viscoelastic medium [28], [17].

The computational approach presented here has been applied to a model of single cell migration and chemotaxis. It is of course of great interest how populations of cells migrate and interact with their micro-environment and each other. To apply the approach used here would be a major challenge if many cells were to be simulated given the fitted nature of the bulk mesh covering the highly dynamic extracellular region. We think the approach presented here is best suited to obtaining detailed information from simulations of single cells or the interaction of a small number of cells. This information can then be used to properly inform population models based on partial differential equations for the density of cells using upscaling techniques.

Ultimately, we would like to extend our approach to model cell migration and chemotaxis in three dimensions. Using a suitable variational formulation, the adaptive moving mesh method used here to evolve grid nodes in the tangential direction should extend to two-dimensional surfaces. This equation system could then be coupled to a geometric evolution law for the surface normal velocity. The MMPDE approach could then be used to generate evolving meshes for three-dimensional bulk domains. The ALE surface finite element method has already been utilised for solving PDEs on evolving surfaces [14], [15] and the bulk ALE–FEM method proposed here also extends naturally to three dimensions. The main challenge in three dimensions however is not likely to be the development of efficient computational techniques but the increased complexity of modelling the interaction of migrating cells with their extracellular environment. The insights gained from modelling attempts in two dimensions would therefore be an essential first step in this direction.

## Figures and Tables

**Table 1:** Non-dimensional default parameter values for cell migration simulations. Parameters for the pseudopod-centred cell migration model are taken from [40].

Quantity	Symbol	Value
Decay rate of activator	*r*_*a*_	2 × 10^−2^
Basic production rate of activator	*b*_*a*_	1 × 10^−1^
Saturation of activator autocatalysis	*s*_*a*_	5 × 10^−4^
Diffusion coefficient of activator	*D*_*a*_	4 × 10^−7^
Production & decay rate of global inhibitor	*r*_*b*_	3 × 10^−2^
Diffusion coefficient of global inhibitor	*D*_*b*_	4 × 10^−5^
Production rate of local inhibitor	*b*_*c*_	7 × 10^−3^
Decay rate of local inhibitor	*r*_*c*_	1.3 × 10^−2^
Diffusion coefficient of local inhibitor	*D*_*c*_	2.8 × 10^−6^
Michaelis–Menten constant	*s*_*c*_	2 × 10^−1^
Scaling of protrusive velocity	*K*_*prot*_	1 × 10^−5^
Cortical tension factor	*λ*_0_	2 × 10^−6^
Cortical tension factor	*β*	2 × 10^−2^
Bulk ligand diffusion coefficient	*D*	10
Ligand association rate	*k*_1_	$\frac{1}{30}$
Ligand disassociation rate	*k*_−1_	1
Ligand–receptor complex diffusion coefficient	*D*_*s*_	1 × 10^−6^
Total number of receptors on cell	2*πr*_0_*R*_*tot*_	7 × 10^4^
